# Efficacy of green tea ( *Camellia sinensis Linn*) 3% extract cream on
 improvement of striae distensae

**DOI:** 10.12688/f1000research.142199.1

**Published:** 2024-03-21

**Authors:** Sartika Ayuningsih, Nelva Karmila Jusuf, Imam Budi Putra

**Affiliations:** 1 Dermatology and Venereology, University of Sumatera Utara, Medan, North Sumatra, Indonesia

**Keywords:** striae distensae, stretch mark, INA score, green tea extract, Camellia sinensis linn

## Abstract

**Background:**

Striae distensae (SD) is a skin condition that frequently causes dermatological consultations and although asymptomatic, it may can cause itch and burning sensation. Green tea extract contains polyphenol, including flavanol, flavandiol, flavonoid, phenolic acid, amino acids and minerals which play a role in the repair of stretch marks through anti-inflammatory mechanism, increase collagen production, fibroblast proliferation, and skin hydration.

**Objective:**

To determine the efficacy of green tea extract cream on striae distensae.

**Methods:**

This is a pre-experimental clinical trial with a pretest-posttest design on 36 subjects with striae distensae. Diagnosis establishes through history taking and clinical evaluation. Imam Nelva Alviera (INA) score was used as SD severity before and after the application of the 3% green tea extract cream carried out at weeks 0, 2, 4, 6, and 8. Side effects and subjects’ satisfaction were also recorded. Cochran test was carried out to see the difference before and after treatment, with a p-value <0.05 considered significant.

**Results:**

Majority of study subjects were 18–25 years (77.8%), had history of pregnancy (75%), had a history of menarche at the age of 12 years (27.8%) and all subjects had striae alba. There was significant decrement in INA score for striae distensae (p<0.001) after eight weeks administration of 3% green tea extract cream. Clinical improvement and no side effects were also noted. All subjects were satisfied.

**Conclusions:**

The use of 3% green tea extract cream can improve the appearance of SD.

## Introduction

Striae distensae or known also as stretch marks are skin condition that causes frequent dermatological consultations. Striae distensae (SD) appear as fusiform or linear lesions with certain length and witdh. ^1^ The main etiology of SD remains unclear and are common in different conditions, for example pregnancy, weight changes and adrenocortical excess. The prevalence of SD is estimated at 40-70% in adolescents. ^1^^,^^2^

Treatment of striae distensae become great challenges because often the current available treatments did not provide satisfactory result. The aim of SD is to induce dermal collagen production, fibroblastic activity, such as to improve tissue strength, increasing skin elasticity, and skin hydration. Several treatments that have been known for the treatment of SD include topical tretnoin, laser, radiofrequency, microdermabrasion, and platelet rich plasma. ^3^^,^^4^

Over decades, natural products to improve skin condition have been developed. In Indonesia, plant extracts are used as medicine. Green tea gains its popularity amongst scientists because of variety health benefits. ^5^^,^^6^ Green tea (Camellia sinensis Linn) contains catechins that have significant effects for health because of their anti-inflammatory and antioxidant activities. There are various types of catechin such as epicatechin (EC), epicatechin gallate (ECG), epigallocatechin (EGC), dan epigallocatechin gallate (EGCG) that may also help in skin regeneration. Catechin may induce fibroblast proliferation, epithelialization, and collagen synthesis. Study by Hajiaghaalipour *et al*. found the use of Camellia sinensis Linn extract may increase cell proliferation, angiogenesis and collagen development. ^7^^–^^9^ Essential fatty acids in green tea extract, such as linoleic acid, oleic acid and tocopherol have the potential to help increasing skin hydration, skin elasticity, and antioxidant. ^10^ This study aimed to find the efficacy of green tea extract in striae distensae.

## Methods

This study is a pre-experimental study with a pretest–posttest design carried out from December 2022 to March 2023 at the Department of Dermatology and Venereology, Prof. dr. Chairuddin Panusunan Lubis, Universitas Sumatera Utara Hospital. All subjects participated in this study had signed informed consent and this study was conducted in accordance with the Declaration of Helsinki. This research was conducted after obtaining approval from the health research ethics committee No:1254/KEPK/USU/2022 obtained from the University of North Sumatra Research Ethics Committee .

The minimum sample size in this study was 36 people.

Green tea was obtained from the Sidamanik green tea plantation, North Sumatra, then the process of making 3% green tea extract cream is carried out at the Medicinal Plant Research and Development Laboratory of the Indonesian Herbal Traditional Medicine Association, Medan.

The participants of this study were 36 women with striae distensae, aged 18–35 years, who came to the outpatient clinic of the Department of Dermatology and Venereology, Prof. dr. Chairuddin Panusunan Lubis, Universitas Sumatera Utara Hospital, Indonesia. All participants were evaluated through history taking and clinical examination. Participants were excluded from the study if they are pregnant, with history of pregnancy, and were on oral, topical, or intervention treatment for striae distensae within last month. Subjects were also excluded from the study if they did not use the cream for three consecutive days and the total use of the cream was less than seven weeks.

Subjects were asked to apply one fingertip unit of cream on striae distensae lesion in each femur every morning and night, and being evaluated every two weeks (weeks 2, 4, 6 and 8), research subjects were asked to come back to have the Imam Nelva Alviera (INA) score calculated, and document SD lesions as well as assessing side effects that occurred during research period. Striae distensae severity was measured using INA (Imam, Nelva, Alviera) score, ^20^ assessment of striae distensae includes the number of lines, the size of the longest striae distensae line, color, and the presence or absence of itching. The INA score were assessed before and after using the green tea extract cream. The questionnaire can be found as *Extended data.* ^27^

Data collected from weeks 0, 2, 4, 6, and 8 were analyzed using SPSS version 22.0 software. They were analyzed using the Shapiro-Wilk normality test. The Cochran test was used to compare the INA score before and after the application of green tea extract cream, with p-value <0.05 was considered significant.

## Results

In this study, all research subjects completed all follow-up until the 8th week. ^26^ The majority of subjects with age range of 18–25 years (77.8%) ( Table 1), with the youngest age was 18 years and the eldest at 32 years. Most of the subjects menarch age was 12 years (27.8%) ( Table 2), with family history of SD (75%) ( Table 3), and all of the subjects were presented with striae alba.

**Table 1.** Distribution of subjects by age.

Age (years)	n	%
18–25	28	77.8
26–35	8	22.2
**Total**	**36**	**100**

**Table 2.** Distribution of subjects by menarche age.

Menarche age (years)	n	%
10	1	2.8
11	3	8.3
12	10	27.8
13	9	25.0
14	7	19.4
15	6	16.7
**Total**	**36**	**100**

**Table 3.** Distribution of subjects by family history.

Family history	n	%
Yes	27	75.0
No	9	25.0
**Total**	**36**	**100**

After the data were collected, the Shapiro-Wilk test was conducted to show the normality of the data and Cochran test was carried out to assess the comparison of stiriae distensae severity with INA score before and after the application of green tea extract cream. At the beginning of the study, there were 31 subjects (86.1%) with severe striae distensae. After using green tea extract cream for eight weeks, a significant decrease in subjects with severe striae distensae became 15 subjects. The p-value obtained through the test is <0.001 ( Table 4). The McNemar test also showed that from the beginning of the study towards the end of study, there was a significant improvement in striae distensae severity from the beginning of study towards week 4, week 6, and week 8. ( Table 5). In this study, the 3% of green tea extract cream did not cause any side effects and all of the participants were satisfied.

**Table 4.** Severity of striae distensae with INA Score.

Time	INA Score	n	(%)	p
Week 0	Mild	0	0	<0.001 *
	Moderate	5	13.9	
	Severe	31 *	86.1	
Week 2	Mild	0	0	
	Moderate	8	22.2	
	Severe	28	77.8	
Week 4	Mild	0	0	
	Moderate	13	36.1	
	Severe	23	63.9	
Week 6	Mild	0	0	
	Moderate	18	50	
	Severe	18	50	
Week 8	Mild	0	0	
	Moderate	21	58.3	
	Severe	15 *	41.7	

* Cochran test.

**Table 5.** Difference in striae distensae severity improvement from week 0 to week 8.

Time	Week 2	Week 4	Week 6	Week 8
Week 0	0.250	0.031 *	0.008 *	0.002 *
Week 2		0.250	0.063	0.016 *
Week 4			0.500	0.125
Week 6				0.500
Week 8				

* Posthoc Test.

## Discussion

Striae distensae may happen in several age range, but the prevalence show an increasing trend starting from adolescents. ^11^ The majority subjects in this study were in the age group of 18-25 years old. This study is in accordance with Putra *et al*. study in 155 subjects with striae distenesae, the mean age was 19 years old. ^12^ In a study by Amal *et al*. with 72 health workers with striae distensae, most of the subjects were with the age range of 18-25 years (93.1%). ^13^

Menarche age in majority of subjects were 12 years. A study by Marques, Madeira and Gama showed that the mean age for menarche was 12.4 years. ^14^ Menarche show hormonal maturation from hypothalamic pituitary ovarian (HPO). One of the hypotheses of striae formation was the estrogen influence as the result of HPO axis maturation. Estrogen may decrease adhesion between collagen fibers and cause the striae lesion. Aryunisari *et al*. concluded that early age of menarche increases the risk of striae formation. ^15^^,^^16^

Majority of the participants in this study do have family history of striae distensae. This study is in accordance with Sipahutar *et al*. study in 202 women with striae distensae, 83.7% had family history of striae distensae. ^17^ Gosh *et al*. also showed than 73.3% subjects with SD have family history of striae distensae. ^18^ It was assumed that there were gene expression involvement in fibroblast metabolism that code elastin, collagen, and fibronectin was lower compared to normal skin. ^19^

There is no single therapy that is truly effective in treating striae distensae. This research is a preliminary study on the use of green tea extract to treat striae distensae. The result of this study showed that 3% green tea extract cream decrease striae distensae severity that was scored with INA score from the beginning of the treatment towards the eight weeks of the treatment. INA score was the newest score that can assess striae distensae severity more specific. The INA score was differentiated into 3 categories, such as mild (<3), moderate (3–6), and severe (>6). ^20^

Striae distensae treatment aim to decrease redness, edema, irritation from striae rubrae, increasing collagen production and elastin fibers, to help in collagen arrangement, increase hydration and decreasing inflammation in striae alba. Epigallocatechin gallate (EGCG) in green tea was proven to increase the expression of transforming growth factor β1 (TGF-β1) in fibroblast proliferation stimulation and regeneration of collagen tissue by inducing collagen and elastin. ^9^ It was suggested that green tea had about twenty times more powerful antioxidant than vitamin E. Study by Yaghmayei *et al*. showed than Camellia sinensis Linn extract can accelerate inflammation phase because of the antioxidant effect in the formation of collagen synthesis and increasing fibroblast proliferation. ^21^^,^^22^ The EGCG in green tea can increase the regulation of klotho gene expression in normal epidermal keratinocytes by protein kinase A (PKA)-cAMP responsive element-binding protein (CREM) signalling, that cause differentiation of keratinocytes. Epigallocatechin gallate mediate TGF-β1 to induce collagen contraction in fibroblasts by suppressing myofibroblast differentiation and decreasing the gene expression from connective tissue growth factor and collagen type I gene. ^23^

Reactive oxygen species (ROS) can cause damaging effect on cell, tissue, and interfere with wound healing process. Antioxidant or free radical scavengers play roles in wound healing by accelerating the healing time, improving the appearance of healed tissue, and at the same time protecting the tissue from oxidative damages. Green tea may aid in wound healing because of its phytoconstituents which is rich in flavonoid and phenolic and high free radical scavenging activity. ^9^

Safety profile of green tea extract has been mentioned in several studies, including a study by Meethama *et al*. in 20 participants who were given green tea extract in different concentration as 2%, 4.5%, and 7% didn’t show any skin irritation. Kim *et al*. also reported that green tea extract may be used as anti-inflammation without toxicity effect. ^24^^,^^25^

In this study there are still limitations where not all confounding factors could be excluded in the research subjects. Therefore, further research with a randomized controlled trial design is needed to further evaluate the efficacy of green tea extract against striae distensae.

## Conclusion

The use of 3% green tea extract cream can improve the appearance of striae distensae and the use of this extract showed there are no side effects and all of the subjects were satisfied. This study shows that green tea extract cream was effective and can be considered as striae distensae treatment.

## Data Availability

Zenodo: Data collected. https://doi.org/10.5281/zenodo.8427287. ^26^ Zenodo: Data set side effect and satisfaction subject. https://doi.org/10.5281/zenodo.8418988. ^27^ This project contains the following extended data:
- Questionnaire Data are available under the terms of the Creative Commons Attribution 4.0 International license (CC-BY 4.0).
