# Supplementary material for: Efficacy of green tea ( Camellia sinensis Linn) 3% extract cream on : improvement of striae distensae
Source: F1000Res. 2024 Mar 21;13:208. [Version 1] doi: 10.12688/f1000research.142199.1 (PMC11157189; doi:10.12688/f1000research.142199.1)
Supplement: Peer review history

# Reviewer report: Reviewer response for version 1

**DOI:** 10.5256/f1000research.155713.r262329

**Published:** 2024-06-06

**Authors:** PRASETYADI MAWARDI

**Affiliations:** 1 Sebelas Maret University/Dr Moewardi General Hospital, Surakarta, Indonesia

The use of green tea cream (Camellia sinensis Linn) 3% extract cream) could be an alternative treatment for Striae distensae (SD) in the future. What is interesting is that researchers used the INA score parameters developed by researchers to assess the effectiveness of SD treatment. It's just that the INA score parameter assessment methodology is not explained adequately in the manuscript, how to minimize the subjectivity of the INA score measurement. If carried out by two or more different assessors, it is not explained in the statistical analysis what the Cohen's kappa value of the assessment is.

 Presentation of research results should be added in the form of graphs or pictures, so that it does not give a monotonous impression, compared to presenting data in tables. So it can make it easier for readers to understand the research results and can also increase the quality of the research. Although research has mentioned several limitations of this study, it is recommended to conduct a randomized control study and the use of more objective parameters in assessing clinical and histologic improvement in the treatment of SD.

Is the work clearly and accurately presented and does it cite the current literature?

Yes

If applicable, is the statistical analysis and its interpretation appropriate?

Yes

Are all the source data underlying the results available to ensure full reproducibility?

Yes

Is the study design appropriate and is the work technically sound?

Yes

Are the conclusions drawn adequately supported by the results?

Yes

Are sufficient details of methods and analysis provided to allow replication by others?

Yes

Reviewer Expertise:

Dermatology, Cosmetology, Venereology, Skin cancer, dermatopathology

I confirm that I have read this submission and believe that I have an appropriate level of expertise to confirm that it is of an acceptable scientific standard.

## References

[1] : High-resolution epiluminescence colorimetry of striae distensae. *J Eur Acad Dermatol Venereol* .2006;20(3) : 10.1111/j.1468-3083.2006.01426.x 282-7 10.1111/j.1468-3083.2006.01426.x 16503888

# Reviewer report: Reviewer response for version 1

**DOI:** 10.5256/f1000research.155713.r262328

**Published:** 2024-04-26

**Authors:** Yuli Kurniawati

**Affiliations:** 1 Sriwijaya University, Palembang, Indonesia

This research of Phytopharmaca about striae distansae is  one of  a novel study. Green tea extract cream have rich flavonoid, phenolic and High free radical scavenging activity induced colagen production and elastin fibers in striae distensae. The INA score is the new criteria for Severity scoring of striae distensae and will be applicable in daily practice. The strengths of the research are the first novel research using a phytopharmaca green tea extract which play a role in the repair of stretch marks. The second, criteria striae distensae severity was measured using INA ( Imam, Nelva, Alviera) score as a new scoring of SD. The third, the result had improve the appearance of SD. 

 The weaknesses of this research are the study was single arm study without comparation with another treatment. The second, the research didn’t mention how to make the extract cream. And the third, the validation of INA score not yet established. The further research still need with multi-centre study and more sample.

Is the work clearly and accurately presented and does it cite the current literature?

Yes

If applicable, is the statistical analysis and its interpretation appropriate?

Yes

Are all the source data underlying the results available to ensure full reproducibility?

Yes

Is the study design appropriate and is the work technically sound?

Yes

Are the conclusions drawn adequately supported by the results?

Yes

Are sufficient details of methods and analysis provided to allow replication by others?

Yes

Reviewer Expertise:

Dermatology cosmetic and aesthetic

I confirm that I have read this submission and believe that I have an appropriate level of expertise to confirm that it is of an acceptable scientific standard.
